# Draft Genome Sequence of *Atopobacter* sp. Strain AH10, Isolated from a Vaginal Swab from Macaca fascicularis

**DOI:** 10.1128/MRA.01643-18

**Published:** 2019-01-24

**Authors:** Dong-Ho Chang, Hyung Seok Seo, Kyeong Ryang Park, Sung-Ki Lee, Kang-Jin Jeong, Haeyoung Jeong, Byoung-Chan Kim

**Affiliations:** a Metabolic Regulation Research Center, Korea Research Institute of Bioscience and Biotechnology (KRIBB), Daejeon, Republic of Korea; b Department of Biological Science and Biotechnology, Hannam University, Daejeon, Republic of Korea; c Department of Obstetrics and Gynecology, College of Medicine, Myunggok Medical Research Center, Konyang University, Daejeon, Republic of Korea; d The National Primate Research Center, KRIBB, Chungcheongbuk-do, Ochang-eup, Republic of Korea; e Infectious Disease Research Center, KRIBB, Daejeon, Republic of Korea; f Department of Biosystems and Bioengineering, KRIBB School of Biotechnology, Korea University of Science and Technology (UST), Daejeon, Republic of Korea; g Department of Bioprocess Engineering, KRIBB School of Biotechnology, UST, Daejeon, Republic of Korea; University of Rochester School of Medicine and Dentistry

## Abstract

A bacterial strain belonging to the genus Atopobacter was isolated from a vaginal swab from a crab-eating macaque (Macaca fascicularis). Here, we report the draft genome sequence of this strain, AH10.

## ANNOUNCEMENT

The microbes that reside in the vagina are mainly classified as Lactobacillus species (1). Lactobacillus crispatus, Lactobacillus jensenii, Lactobacillus gasseri, and Lactobacillus iners are the species commonly found in healthy women (1–3). Bacterial vaginosis, a dysbiosis of the vaginal microbiota characterized by diverse and anaerobic bacteria, seems to be associated with undesirable delivery outcomes, such as preterm birth (1–3). Because of the difficulties with human studies, primates, such as the crab-eating macaque (Macaca fascicularis), may be used as animal models for studying high-risk pregnancies, including those that result in preterm birth (4). To use M. fascicularis as an animal model for studying high-risk pregnancies, the primate’s vaginal microbiota should first be investigated by applying both culture-dependent and -independent molecular techniques. While investigating the diversity of the vaginal microbiota from M. fascicularis, a strain belonging to the genus Atopobacter was newly isolated in pure culture by more than three rounds of single-colony isolation from a vaginal swab. Anaerobic DSM 104 medium (http://www.dsmz.de/microorganisms/medium/pdf/DSMZ_Medium104.pdf) was used for both isolation and liquid cultivation under strict anaerobic conditions, as previously described (5).

The genomic DNA of Atopobacter sp. strain AH10 was extracted as previously described (6). Sequencing library construction with an insert size of ca. 500 bp, using a TruSeq Nano DNA kit (Illumina, San Diego, CA, USA), and genome sequencing were carried out on an Illumina MiSeq platform at Macrogen (Seoul, Republic of Korea). A total of 2,997,752 paired reads at 2 × 301 cycles, totaling 902.3 Mb (ca. 408.7× genome coverage, 84.59% Q30), were trimmed using Trimmomatic version 0.32 (7), with SLIDINGWINDOW:4:20, MINLEN 75, and all other parameters were adopted from those used in A5-miseq pipeline version 2015022 (8); the resulting 2,526,108 reads with an average length of 268.4 bp (648.1 Mb in total) were *de novo* assembled using the CLC Genomics Workbench version 11.0.1 (Qiagen Bioinformatics, Aarhus, Denmark). Word and bubble sizes were set to 64 and 80, respectively. After discarding contigs under the average read coverage of 6×, 77 contigs with a total length of 2,207,974 bp (38.6% G+C content) were obtained. The *N*_50_ value and the largest contig length were 77,083 bp and 176,155 bp, respectively. Genome annotation was carried out using the National Center for Biotechnology Information (NCBI) Prokaryotic Genome Annotation Pipeline (9) and the Rapid Annotations using Subsystems Technology (RAST) server (10) with the RAST*tk* (11) annotation scheme and default parameters, and the resulting genome annotation was deposited in GenBank. The number of predicted coding sequences (CDSs) was much higher for RAST than for PGAP (2,100 versus 1,958, respectively), because PGAP counts multiple frameshifted CDSs at a single known locus as one pseudogene. The numbers of tRNAs were the same between two annotations.

To the best of our knowledge, this is the first reported publicly available genome sequence of an Atopobacter strain isolated from a primate vagina. Furthermore, this is the second available genome sequence from the genus Atopobacter; the other is that of the type strain Atopobacter phocae CCUG 42358 (GenBank accession number JAGN00000000). The most closely related strain, as suggested by the NCBI Sequence Read Archive (SRA) taxonomy analysis service, was the human vaginal isolate Anaerococcus lactolyticus S7-1-13 (GenBank accession number JRMW00000000), not A. phocae CCUG 42358^T^, and the percent alignment of AH10 to each of these strains was 1.90% for S7-1-13 and 0.10% for CCUG 42358^T^, respectively. BLAST-based average nucleotide identities between AH10 and these two strains, calculated using the ANI.pl Perl script (https://github.com/chjp/ANI), were 73.3% and 67.7%, respectively.

The genome information from strain AH10 improves our understanding of primate vaginal microbial genetic diversity. This knowledge will help us study high-risk pregnancies using M. fascicularis as an animal model, especially with respect to human reproductive physiology.

### Data availability.

The complete genome sequence of Atopobacter sp. strain AH10 has been deposited in DDBJ/ENA/GenBank under the accession number QXPZ00000000. The version described in this paper is version QXPZ01000000. Raw sequencing reads are available in NCBI under BioProject number PRJNA489511.
